# Dataset on force measurements of needle insertions into two ex-vivo human livers

**DOI:** 10.1016/j.dib.2017.01.018

**Published:** 2017-02-15

**Authors:** Tonke L. de Jong, Jenny Dankelman, John J. van den Dobbelsteen

**Affiliations:** BioMechanical Engineering Department, Delft University of Technology, 2628 CD Delft, The Netherlands

## Abstract

A needle-tissue interaction experiment has been carried out, by inserting the inner needle of a trocar needle into two ex-vivo human livers. The dataset contains the forces that act on the needle during insertion and retraction into the livers. In addition, a MATLAB code file is included that provides base-level analysis of the data and generates force-position diagrams of the needle insertions. The dataset is available on Mendeley Data (do1i:10.17632/94s7xd9mzt.2), and is made publicly available to enable other researchers to use it for their own research purposes.

For further interpretation and discussion of the data, one is referred to the associated research article entitled “PVA matches human liver in needle-tissue interaction” de Jong et al., 2017.

**Specifications Table**

**Table:** 

Subject area	Engineering
More specific subject area	Medical engineering, needle-tissue interaction experiments
Type of data	Data files in .mat, .m and .fig format
How data was acquired	Force sensor data
Data format	Raw unfiltered data and base-level Matlab code
Experimental factors	Ex-vivo human livers embedded in 10 m% gelatin-to-water
Experimental features	Axial force sensor data of needle insertions into two ex-vivo human livers embedded in 10 m% gelatin-to-water
Data source location	– Data at Biomechanical Engineering Department, Delft University of Technology, 2628 CD, Delft, The Netherlands – Experiments conducted at Neurosciences Department, Erasmus MC, University Medical Center, 3000CA, Rotterdam, The Netherlands
Data accessibility	Data with this article is on Mendeley Data (doi:10.17632/94s7xd9mzt.2)

**Value of the data**

• Experimental needle-tissue interaction data with real human tissue is scarce. This dataset can be useful as reference data for other researchers.
• These data can e.g. be used for the development of medical phantoms, steerable needles and robotic positioning systems.
• This dataset is available for comparison to other needle insertion force data, such as in: diseased human tissue, in-vivo tissue, animal tissue, and tissue mimicking materials.

## 1 Data

This dataset is derived from a needle-tissue interaction experiment, in which the inner needle of an 18 G trocar needle with triangular tip was inserted multiple times into two ex-vivo human livers. Data include for each of the 39 insertions:

– Forces [N] during needle insertion and retraction
– Position [mm]
– Time [s]

## 2 Experimental design, materials and methods

For a detailed description of the experimental set-up, please refer to [1].

### 2.1 Ex-vivo human liver preparation

Two human liver specimens were extracted from fresh-frozen cadavers. These livers were obtained from persons without hepatic cirrhosis, as the anatomy did not show any suspicious nodules. The thickness of both livers was approximately 70 mm. The livers were embedded in 10 m% gelatin-to-water (Dr. Oetker, Bielefeld, Germany), to simulate the abdominal environment. Embedding in gelatin took place within one day after extraction. In the meantime, the extracted livers were stored in water in a plastic box in a refrigerator (±4 °C). The test specimens were created by placing the livers on a gelatin base layer, to enable puncturing through the whole liver. Subsequently, the liver was submerged by a second gelatin solution, to fixate the liver. This gelatin solution was cooled down to 40 °C, to prevent thermal damage of the tissue. Finally, a top gelatin layer was created. The container was stored overnight in the refrigerator to ensure proper stiffening of the gelatin.

### 2.2 Experimental design

During each run, the needle was inserted by the linear motion stage with a constant insertion and retraction velocity of 5 mm/s. The needle was going from the upper gelatin layer through the liver into the lower gelatin layer, so that the entire liver was punctured (approximate height of 70 mm).

The needle insertion locations were predefined and randomized. The livers were each subjected to 20 needle runs, with a mutual distance of at least 10 mm. A new needle was used for each liver.

### 2.3 Dataset

For every insertion and retraction, the axial forces acting on the needle hub were stored, as well as corresponding time and positions, with a sample frequency of 1 kHz. Note that whereas a total of 40 insertion were planned, only 39 were included in the dataset, due to improper saving of the force data of one needle run. The raw, unfiltered data of the insertions into two livers is respectively shown as force-position diagrams in interactive MATLAB Figs. 1 and 2. In addition, base level MATLAB code is included to load the raw data in MATLAB and create the figures.

## Footnotes

Transparency document Transparency data associated with this article can be found in the online version at doi:10.1016/j.dib.2017.01.018.

Appendix A Supplementary document associated with this article can be found in the online version at doi:10.1016/j.dib.2017.01.018.
