# Supplementary material for: Dataset on force measurements of needle insertions into two ex-vivo human livers
Source: Data Brief. 2017 Feb 15;11:308–10. doi: 10.1016/j.dib.2017.01.018 (PMC5498459; doi:10.1016/j.dib.2017.01.018)
Supplement: Supplementary file 3 — Supplementary Fig. 3. [file mmc3.pdf]

Conflicts of Interest Statement

Manuscript title: Dataset of force measurements of needle insertions into two ex-vivo human livers.

The authors whose names are listed immediately below certify that they have NO affiliations with or involvement in any organization or entity with any financial interest (such as honoraria; educational grants; participation in speakers' bureaus; membership, employment, consultancies, stock ownership, or other equity interest; and expert testimony or patent-licensing arrangements), or non-financial interest (such as personal or professional relationships, affiliations, knowledge or beliefs) in the subject matter or materials discussed in this manuscript.

Author names:

Tonke L. de Jong 15 feb 2017

John J. van den Dobbelsteen " "

Jenny Dankelman " 16-2-2017 "

The authors whose names are listed immediately below report the following details of affiliation or involvement in an organization or entity with a financial or non-financial interest in the subject matter or materials discussed in this manuscript. Please specify the nature of the conflict on a separate sheet of paper if the space below is inadequate.

Author names:
